# Enhancement of Diffusion-Controlled Pseudocapacity in Biphasic NaMnO_**2**_ Electrodes for Sodium Batteries by Tailoring Structural and Morphological Properties

**DOI:** 10.1021/acsomega.5c03760

**Published:** 2025-06-25

**Authors:** Andrii Boichuk, Tetiana Boichuk, Marie Kreĉmarová, Mahesh Eledath Changarath, Rafael Abargues, Said Agouram, Juan F. Sánchez-Royo

**Affiliations:** † ICMUV, Instituto de Ciencia de Materiales, 253325 Universidad de Valencia, Valencia 46071, Spain; ‡ King Danylo University, Ivano-Frankivsk 76000, Ukraine; § Department of Applied Physics and Electromagnetism, University of Valencia, Valencia 46100, Spain

## Abstract

In this study, we present a biphasic (orthorhombic/monoclinic) NaMnO_2_ material synthesized by using cheap and low-temperature sol–gel methods, which presents potential applications as an advanced cathode material for aqueous sodium-ion energy storage. By leveraging its unique structural and morphological properties, our approach optimizes the balance between diffusion-controlled and surface-controlled pseudocapacitive charge storage, significantly enhancing electrochemical performance with capacity values over 100 mAh/g. Electrochemical investigations reveal that the tailored morphology of NaMnO_2_ allows for a high pseudocapacitive contribution, with the Na_2_SO_4_ electrolyte exhibiting the most stable cycling behavior and the highest capacity retention. Ex-situ X-ray diffraction, Raman spectroscopy, transmission electron microscopy, and X-ray photoemission techniques confirm both intercalation and surface pseudocapacitive reactions, highlighting the interplay between intercalation and adsorption processes. The calculated value of diffusion-controlled pseudocapacity contribution of about 55% enables superior charge storage capabilities and rapid charge/discharge rates. These findings establish biphasic NaMnO_2_ as a promising, structured, stable electrode material for next-generation, high-performance, and robust sodium energy storage systems.

## Graphical abstract

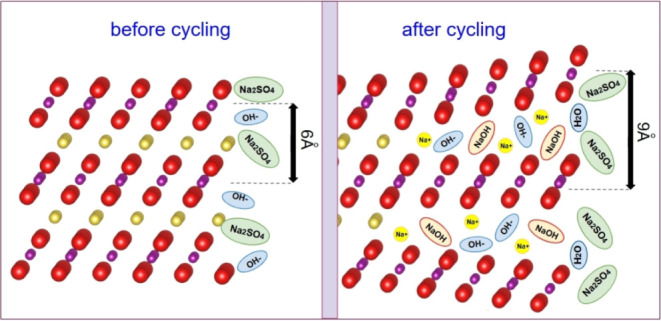

## Introduction

Due to the abundant sodium reserves in the Earth’s crust and the similar electrochemical activity of sodium and lithium ions, sodium-based electrochemical energy storage systems (batteries and supercapacitors) hold significant promise for large-scale energy storage and grid development. With this motivation, most scientific and engineering efforts have focused on developing industrially applicable electrode materials. Vehicle applications require power sources with both high energy and high power, which a Faraday-type electrode (a cathode) can provide.

Layered presodiated manganese oxides are promising electrode materials due to their stable structure and ability to reversibly intercalate and deintercalate sodium ions, resulting in good electrochemical performance.^1–6^ DFT calculations employed in the latest research^7^ to assess the electrochemical properties and stability of monoclinic NaMnO_2_ show a good theoretical reversible capacity. However, possible phase transitions described for the monoclinic structure, triggered by varying sodiation levels due to changes in the manganese oxidation state, may affect the stability of the electrochemical performance. On the other hand, P2-type Na–Mn–O systems^8^ exhibit better cycling performance and rate capability than the O3 phase. Taking these important insights into account, biphasic (orthorhombic and monoclinic) layered NaMnO_2_ can be considered a promising structure, combining good structural stability with high specific capacity and energy density.^9,10^ Despite the high specific indicators and cycling stability (primarily determined by the structure), there remains an unresolved task of optimizing particle size to optimize the contact area with electrolyte and, respectively, allow for rapid electrochemical reactions, which are crucial for operation under high-current conditions. We previously demonstrated success in the sol–gel preparation of biphasic (orthorhombic/monoclinic) NaMnO_2_ with a high degree of crystallization,^11^ allowing for control over particle size and morphology, tailoring them for specific applications. In particular, it is essential to determine the influence of morphology on the intensity and reversibility of electrochemical reactions when using these materials, for example, in cost-effective and safe aqueous batteries and supercapacitors.^12–14^ The specific capacity and power of such electrochemical devices are primarily determined by the cathode. Since cathode materials exhibit a combined capacity resulting from both diffusion and absorption,^15–17^ optimizing the ratio between diffusion-controlled and absorption-controlled reactions is important for achieving high specific electrochemical performance.

By performing cyclic voltammetry (CV) at different scan rates, we can efficiently and rapidly analyze this ratio electrochemically. To explore more, it is necessary to validate these findings through fundamental ex-situ investigations after cycling, allowing us to determine the distribution of intercalated sodium and gather other critical information. The spatial separation of these processes requires the use of techniques such as electrochemical impedance spectroscopy (EIS), Raman spectroscopy, and scanning electron microscopy (SEM) to observe changes on electrode surfaces and in their immediate vicinity (macro level), as well as changes in the structure and morphology of active material particles (micro level) using methods such as X-ray diffraction (XRD), X-ray photoelectron spectroscopy (XPS), and transmission electron microscopy (TEM). There is an evident lack of such a comprehensive research approach in aqueous electrochemical systems, and it is entirely absent in the case of biphasic presodiated manganese oxide with submicron particles. In our view, this combination of studies can provide valuable insights into improving electrochemical properties by optimizing the intercalation/absorption ratio, which can be achieved by modifying the particle morphology. In this study, we compared results from cyclic voltammetry and employed a combination of fundamental ex-situ measurements to verify the distinction between these two types of pseudocapacitance during the operation of biphasic NaMnO_2_ in an aqueous electrochemical system.

## Experimental Section

### Synthesis and Characterization of NaMnO_2_ Samples

Biphasic sodium manganese oxide was prepared using a sol–gel method. As precursors, we used manganese acetate tetrahydrate (Thermo Scientific, 99%) and sodium nitrate (Panreac, 99%) dissolved in water (0.2 M solution) with the final calcination of the obtained gel carried out for 15 hours at 750 °C under an air atmosphere (Figure S1). The samples were characterized using XRD in the range of 10–80° using a Bruker D8 ADVANCE A25 diffractometer (Cu–K radiation source, wavelength λ = 1.54 Å). We performed cyclic voltammetry and electrochemical impedance spectroscopy (EIS) tests using Gamry 5000P equipment in a three-electrode cell with Pt wire and Ag/AgCl (3 M KCl) electrodes as counter and reference electrodes, respectively, in an aqueous solution of 1 M Na_2_SO_4_, Na_2_CO_3_, and NaNO_3_ (100 cycles, scan rate 50 mV/s). NaMnO_2_ (80 wt %) was ground in a mortar with conductive carbon black (15 wt %) and PTFE binder (5 wt %).

High-resolution transmission electron microscopy (HRTEM) images with a field emission gun TECNAI G^2^ F20 microscope operated at 200 kV were obtained for the powder and electrodes after cycling.

XPS measurements were performed in a SPECS GmbH system (base pressure 1.0 × 10^–10^ mbar) equipped with an ASTRAIOS 190 2D-CMOS hemispherical analyzer. Photoelectrons were excited with the Al-Kα line (1486.7 eV) of a monochromatic μ-FOCUS 500 X-ray source (SPECS GmbH).

Raman spectra were obtained using a confocal Raman microscope; Horiba-MTB Xplora measurements were carried out at room temperature using red laser illumination with a 638 nm excitation wavelength, 16 mW laser power, and 50× objective (N.A. = 0.3, WD = 1.3 mm). The optical beam was focused on the sample with a spot size of approximately 1 mm^2^ and a power of 100 mW.

### Electrochemical Measurement Technology

Electrodes were prepared by applying multiple layers of the mixture onto nickel foam, which was ultrasonically cleaned in acetone and deionized water for 10 min each and thoroughly dried at 80 °C. The active material (80 wt %) was ground in a mortar together with 15 wt % conductive carbon and a 5 wt % PTFE binder. The mixture was then homogenized using an Ultra-Turrax until the slurry was achieved. A soft, fine-bristled brush was used for coating, with the slurry applied slowly in one direction under steady pressure. CVA tests (scan rates of 0.5, 2, 5,10, 20, and 50 mV/s) and charge–discharge cycling were conducted using Gamry 5000P equipment in a three-electrode cell, where a Pt wire served as the counter electrode and an Ag/AgCl electrode as the reference,^18^ in an aqueous solution containing 1 M Na_2_CO_3_, Na_2_SO_4_, and NaNO_3_. Ex-situ XRD, XPS, Raman, SEM, and TEM studies were conducted for electrodes after CVA cycling (100 cycles at 50 mV/s). EIS for the three-electrode cells was also performed using the same equipment before and after 100 cycles of CV at 50 mV/s in different electrolytes. The measurements were taken at open-circuit voltage in a frequency range from 20 kHz to 0.01 Hz (63 points) with an alternating current voltage amplitude of 5 mV.

## Results and Discussion

CVA curves of three-electrode cells recorded at a scan rate of 50 mV/s for different electrolytes (1 M solutions of NaNO_3_, Na_2_SO_4_, and Na_2_CO_3_) are presented in Figure 1. During the first 100 cycles, we observe completely different behavior in the curves, especially in the high-voltage region, along with changes in their shape. For all electrolytes, in the first cycle, the CVA curve exhibits a typical shape associated with pseudocapacitive charge accumulation without sharp intercalation/deintercalation peaks. For the NaNO_3_ electrolyte (Figure 1a), after 25 cycles, an increase in the closed area of the CVA curve is observed.

**Figure 1:**
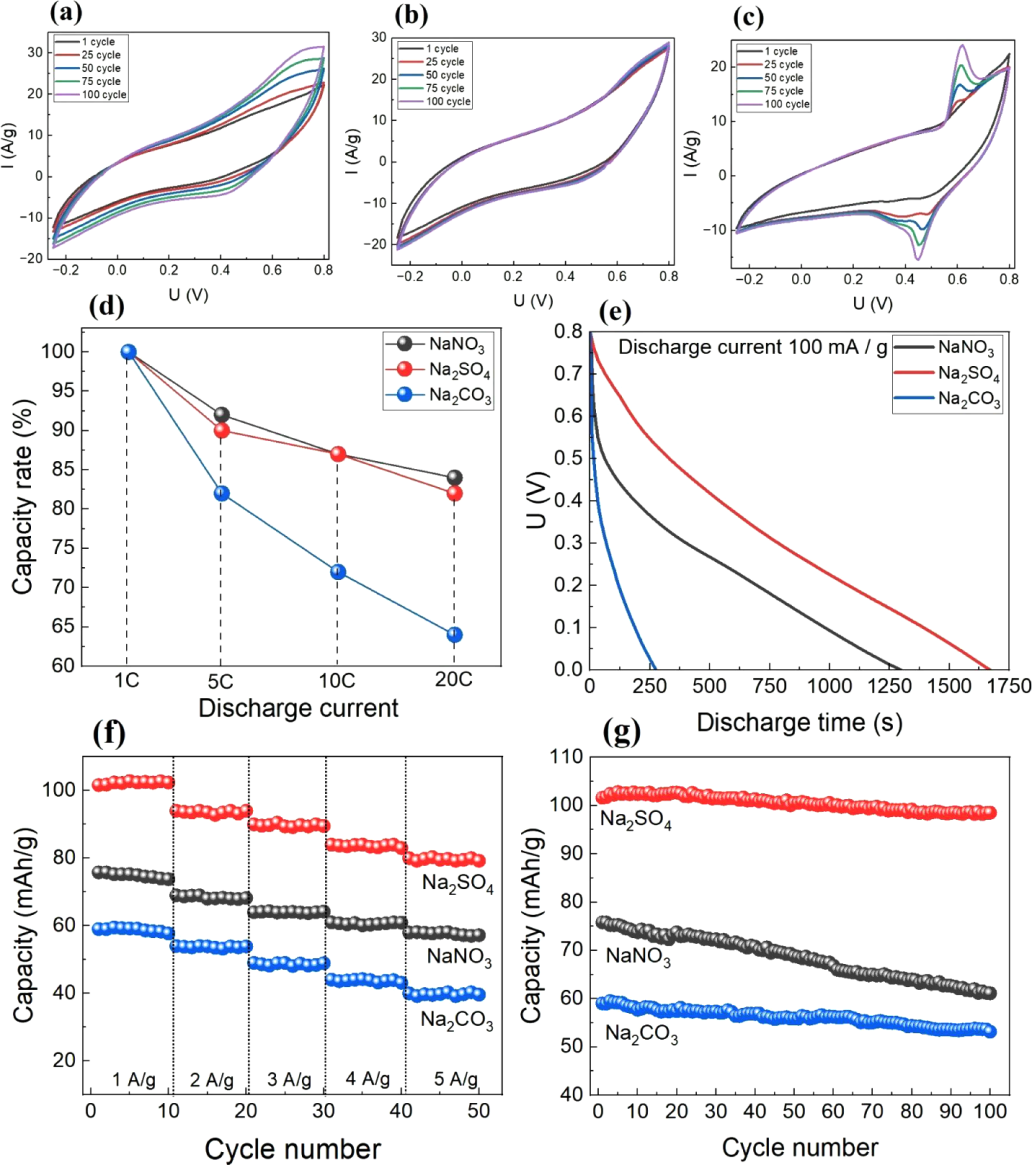
100 CVA cycles of three-electrode cells in different electrolytes, (a) NaNO_3_, (b) Na_2_SO_4_, and (c) Na_2_CO_3_ at a scan rate of 50 mV/s; capacity rate (in percent compared with capacity at 1C) at different currents (5C, 10C, and 20C) for three-electrode cells based on NaMnO_2_ (d) and discharge curves of different electrolytes (e); capacity at different discharge current rates (f) and capacity retention during 100 cycles under current 1A/g (g) for all electrolytes.

At the same time, in the Na_2_SO_4_ electrolyte (Figure 1b), the CVA behavior remained the most stable during cycling. In the case of Na_2_CO_3_, after the 25th cycle, redox peaks appeared, with their intensity increasing over continued cycling. The observed differences in CVA behavior among these electrolytes could be attributed to the following factors: (i) electrolyte properties (e.g., resistance (conductivity), reactivity), (ii) changes at the electrode/electrolyte interface during cycling, and (iii) structural changes in the electrodes over cycling. We discarded the first factor, as it does not affect the structural properties of the electrode materials. Rather, the stability of the biphasic NaMnO_2_ structure and the electrode/electrolyte interface plays a key role in achieving good electrochemical performance, particularly in terms of power density, which reflects the ability to maintain optimal operating values at high current rates. Based on the discharge curves of the three-electrode cell (Figure 1e), the best performance is observed for the Na_2_SO_4_ electrolyte, while the Na_2_CO_3_ solution exhibits the shortest discharge time during cycling. An increase in discharge current (Figure 1d) from 1C to 20C leads to a decrease in capacity retention: for the Na_2_CO_3_ electrolyte, capacity retention is about 64%, whereas for the other two electrolytes, it remains higher, reaching up to 85%. Charge–discharge cycling under different current rates (Figure 1f) demonstrates good capacity retention across all electrolytes (10 cycles at each current), indicating the high reversibility and cyclability of the electrodes. Moreover, after 100 charge–discharge cycles (Figure 1g), we observed capacity retention of approximately 96% in Na_2_SO_4_, around 80% in Na_2_CO_3_, and about 87% in NaNO_3_. These results are consistent with the stability of CVA curves’ shape: the highest capacity and capacity rate occur during cycling in Na_2_SO_4_, and the lowest in Na_2_CO_3_. Moreover, we did not observe a horizontal plateau on discharge curves in Na_2_CO_3_, so an intercalation of sodium ions process does not seem to be behind the observed CVA cathodic peaks at 0.44–0.47 V (Figure 1c). Also, for NaNO_3_, we observed a significant increase of the current peak in the anodic direction (end of charge) from 20 A/g at the first cycle up to 33 A/g at the 100th cycle. Despite the correlation of calculated operating values with CVA/discharge results, we observed clear differences in mechanisms of pseudocapacitive charge accumulation: different contributions from diffusion-controlled and surface reactions lead to different performance of electrodes. To quantify this contribution for the electrode material based on NaMnO_2_ in different electrolytes, except for the electrochemical techniques, we performed fundamental research on the structure and morphology of electrodes during operation.

Ex-situ XRD results after 100 cycles in different electrolytes (Figure 2a–c, red line) show that the peak intensities of the cycled electrodes have no pronounced peak shifts or new Bragg reflections in comparison to the pattern of NaMnO_2_ powder (black line). Additional high-intensity peaks located around 44, 52, and 77 degrees related to the nickel foam^19^ (current collector for electrodes) and carbon black (the one located at ∼26 degrees) have been observed.^20^ We did not observe a significant shift of the (001) peaks of orthorhombic (insets in Figure 2a–c) and monoclinic structures in our biphasic electrode material after 100 cycles. These XRD results provide evidence of the stability of the crystal structure after cycling, at least in the core of NaMnO_2_ particles. However, these studies do not give clear evidence about the intercalation processes of sodium ions taking place in electrodes or even eventual changes occurring at their surface as a consequence of cycling.

**Figure 2:**
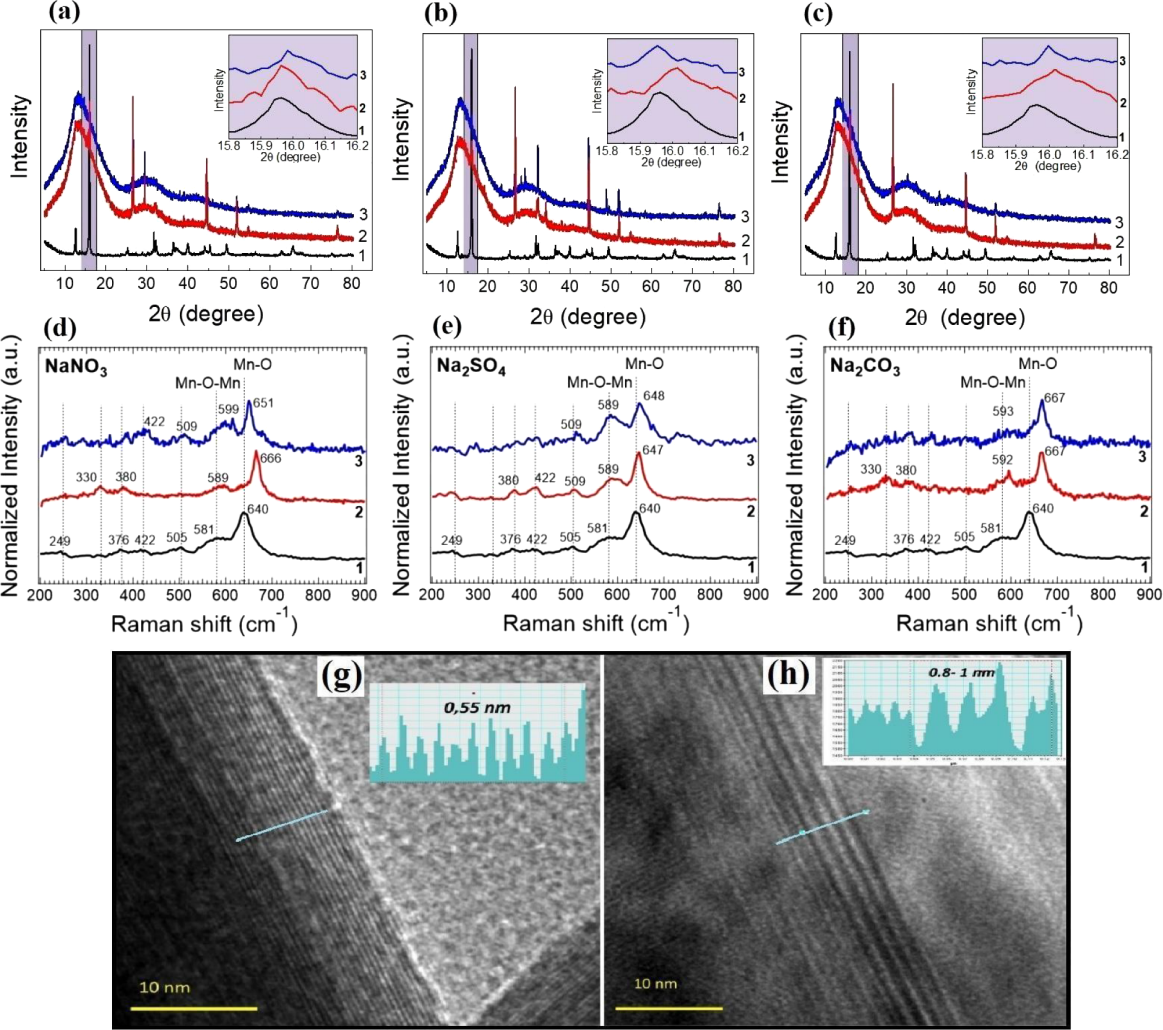
XRD and Raman spectrum comparison of NaMnO_2_ in 1 M NaNO_3_ (a,d), Na_2_SO_4_ (b,e), Na_2_CO_3_ (c,f) electrolytes, respectively (for 1: pristine NaMnO_2_ sample, 2: after its cycling, and 3: after dipping in the corresponding electrolyte without cycling). HRTEM images of NaMnO_2_ sample before (g) and after (h) cycling in Na_2_SO_4_.

Therefore, an additional advanced characterization technique should be used to reveal the charge storage mechanism and separate bulk intercalation and surface adsorption. Raman spectroscopy techniques can shed light on these questions. Figure 2d–f shows Raman spectra acquired from pristine NaMnO_2_ samples exposed to ambient air and soaked in 1 M NaNO_3_, Na_2_SO_4_, and Na_2_CO_3_ electrolytes for 12 h. Raman spectra of the pristine sample (Figure 2d–f, curve 1) exhibit two strong peaks located at 581 and 640 cm^–1^ and four weak peaks located at 249, 376, 422, and 505 cm^–1^ representing Mn–O and Mn–O–Mn lattice vibration modes within the MnO_6_ octahedral framework.^21–25^ The presence of the strong highest-frequency bands indicates a well-developed tetragonal structure.^22,26^ The peak located at 581 cm^–1^ is attributed to Mn–O vibrations along the MnO_6_ octahedral chains, and the peak at 640 cm^–1^ to Mn–O vibrations perpendicular to the direction of MnO_6_ octahedral double chains.^22,26^ After the samples were dipped or cycled in the three different electrolytes NaNO_3_ (Figure 2d), Na_2_SO_4_ (Figure 2e), and Na_2_CO_3_ (Figure 2f), the most evident detected change is a blue shift of the two highest-frequency Mn–O vibration modes previously located at 581 and 640 cm^–1^ (in the pristine sample). The detected blue shift might suggest a partial crystal structure change (different lengths of Mn–O bonds)^26^ after sample contact with electrolytes. Among other reasons, this shift can be caused by a higher oxidation state of Mn (e.g., increased Mn^4+^ content), which generally results in stronger Mn–O bonding during the charge–discharge process, particularly during sodium deintercalation (oxidation). Other contributing factors may include adsorption of electrolyte species and mechanical strain induced by cycling. Depending on the type of electrolyte used in this study, the highest sample stability (after both dipping and cycling) corresponds to devices using Na_2_SO_4_ electrolytes (these showing the lowest blue shift of the Raman modes at ∼8 cm^–1^). Conversely, our results indicate that electrodes in NaNO_3_ or Na_2_CO_3_ electrolytes exhibit lower stability, as they show a larger blue shift of Raman modes from ∼ 8 to ∼26 cm^–1^ and from ∼11 to ∼27 cm^–1^ after cycling/dipping, respectively.

The modification of the electrode surface structure due to cycling processes has been studied by HRTEM (Figure 2g,h). These results reveal that the intralayer structure of NaMnO_2_ electrode materials remains unaltered after cycling, since lattice spacing values are similar to those obtained in the as-prepared NaMnO_2_ ^11^ (see Table S1). On the contrary, as we can extract from the HRTEM results depicted in Figure 2h and Table S1, we observe an increase of the (001) interlayer spacing up to 0.8–1.0 nm as compared to the (001)-plane spacing measured in the monoclinic/orthorhombic structure. These results evidence the structural evolution in the near-surface region of initial NaMnO_2_ particles, which evolve from monolayers before cycling to stacked layers after 100 cycles. Based on EDAX analysis in nanoprobe mode (Figure S2 and Table S2), the Mn:Na ratio decreased from 2.36 before cycling to 2.11 after cycling. A higher amount of Na between Mn–O layers may indicate intercalation of Na^+^, OH^–^, or NaOH, which is a plausible hypothesis taking into account the increase of interlayer spacing observed after cycling. SEM images of NaMnO_2_ particles (Figure 3a) show a small size of about 1 mm and a typical layered structure of the material. The surface of the electrode after mixing with carbon black and binding agent before cycling is presented in Figure 3b. After 100 cycles in different electrolytes (Figure 3f–h), the electrodes maintain good geometric integrity without any cracks or breaks, indicating good stability during operation.

**Figure 3:**
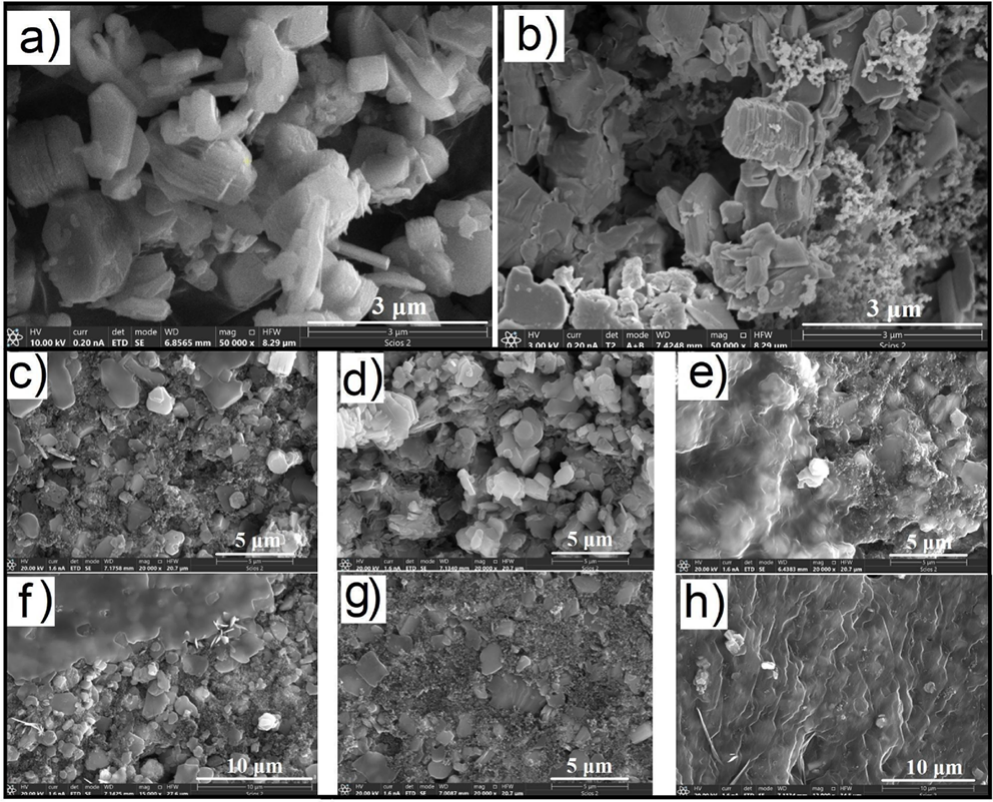
SEM images of NaMnO_2_ powder (a); NaMnO_2_-based electrodes with carbon black before cycling (b); cycled electrodes (c–e) and dipped for 12 h (f–h) in NaNO_3_ (c,f), in Na_2_SO_4_ (d,g) and Na_2_CO_3_ (e,h) electrolytes.

To distinguish between the effect of electrode contact with the electrolyte and the effect of cycling on its surface properties, SEM and XPS spectra were obtained for dipped and cycled electrodes. As we can see in Figure 3c–e for different electrolytes, the formation of a low conductive film occurs in different ways, even before cycling (after dipping in the electrolyte for 12 h). For instance, in the case of the Na_2_CO_3_ electrolyte, the surface of the electrode is totally covered by this film, and during cycling (Figure 3h), the thickness of this film increases, which is caused by the highest solvation energy of the CO_3_^2–^ anion and the formation of a solid electrolyte interface on the surface of the electrodes. For NaNO_3_ and Na_2_SO_4_, interfacial film formation occurs after cycling with lower thickness and a non-homogenized distribution of elements (Figure S3).

X-ray photoelectron spectroscopy (XPS) was employed to elucidate the chemical states of elements composing the NaMnO_2_ electrodes in three different states: (i) in their pristine state, (ii) after 12 h of immersion in the different electrolytes (NaNO_3_, Na_2_SO_4_, and Na_2_CO_3_), and (iii) after cycling. Figure 4a,d,g depicts the deconvoluted Na 1s core-level XPS spectra acquired from the NaMnO_2_ electrodes in each of the different states mentioned above. For the bare electrode, its Na 1s core-level spectrum exhibits a single peak centered at 1072.1 eV,^27,28^ which corresponds to the Na^+^ ion within the NaMnO_2_ lattice. Upon immersion in NaNO_3_ for 12 h (Figure 4a), the Na 1s spectrum measured in the electrode reveals three distinct components: the one related to the NaMnO_2_ lattice already observed in the pristine electrode, although downshifted by 0.8 eV with respect to that one; a second one attributed to metallic sodium (Na^0^) located at 1069.1 eV; and a third one attributed to NaNO_3_ centered at 1072.7 eV.^29^ After cycling in NaNO_3_ (Figure 4a), the Na 1s core-level spectrum measured in the electrode retains the peaks associated with NaMnO_2_ and NaNO_3_. Although a new additional component can be detected at 1074.2 eV, this can be attributed to a NaOH layer formed during cycling and located between layers of electrode material and/or on the electrode surface.^11^

**Figure 4:**
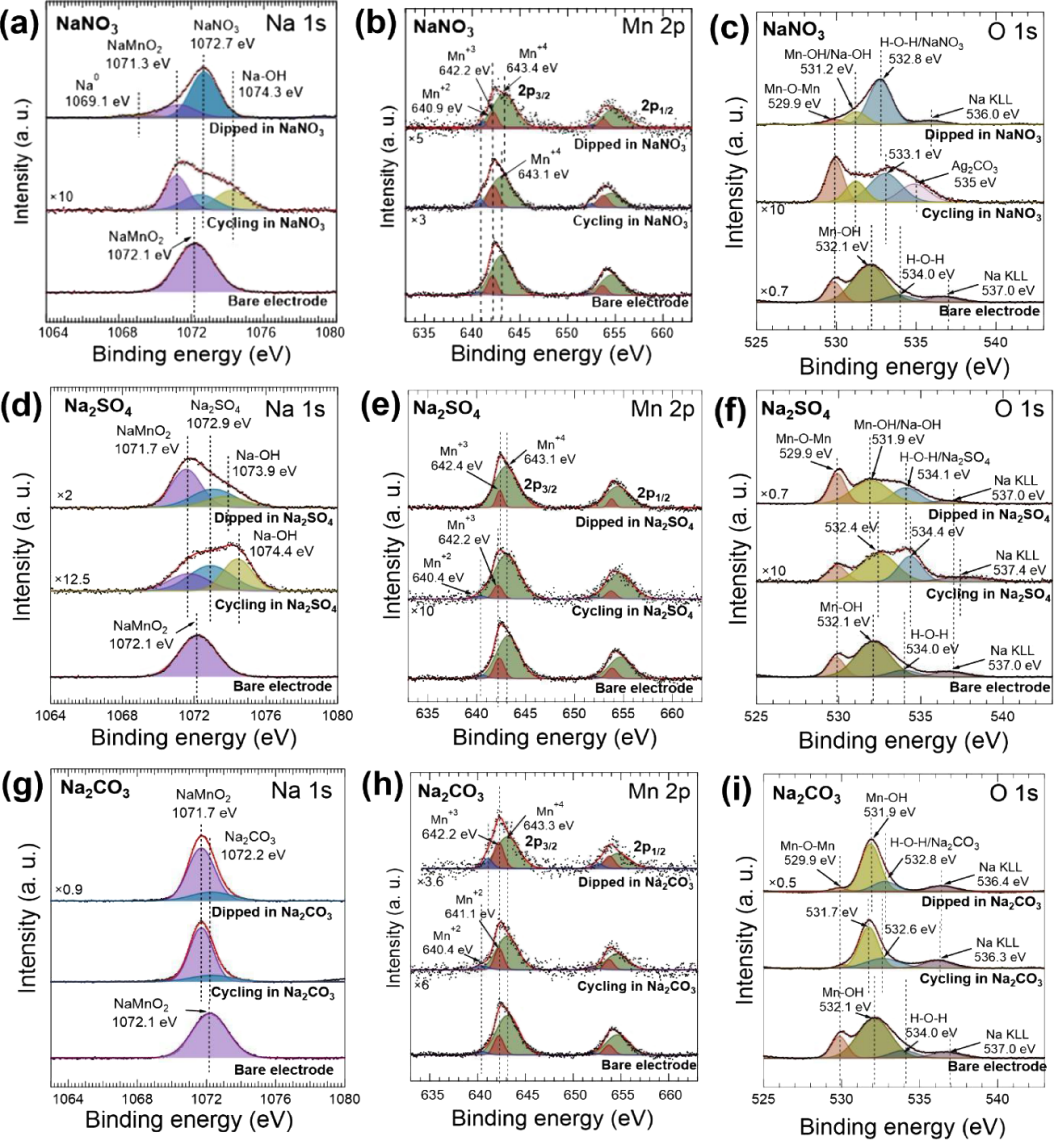
Na 1s, Mn 2p, and O 1s core-level spectra acquired by XPS in NaMnO_2_ samples under different operation states: as prepared, dipped for 12 h in electrolytes, and after cycling. The spectra were acquired in electrodes dipped and cycled in NaNO_3_ (a–c), Na_2_SO_4_ (d–f), and Na_2_CO_3_ (g–i) electrolytes.

The Na 1s spectrum acquired from the electrode after dipping in Na_2_SO_4_ (Figure 4d) shows three peaks, which are attributed to NaMnO_2_ (1071.6 eV), Na_2_SO_4_ (1072.5 ± 0.4 eV),^30,31^ and NaOH (1073.8 eV).^28^ Similarly, the spectrum of the electrode after cycling in Na_2_SO_4_ maintains these three peaks, indicative of the presence of such compounds. For electrodes immersed or cycled in a Na_2_CO_3_ solution (Figure 4g), the deconvolution of the Na 1s core-level spectra reveals two distinct peaks, corresponding to NaMnO_2_ (1071.7 eV) and Na_2_CO_3_ (1071.8 ± 0.4 eV).^32,33^ Notably, the NaMnO_2_ peak shifts to a lower binding energy in both the immersed and cycled samples compared to that in the pristine electrode, strongly suggesting the intercalation of Na^+^ ions from the electrolyte into the layered NaMnO_2_ lattice. Figure 4b,e,h presents the Mn 2p core-level XPS spectra collected from the pristine and electrolyte-treated NaMnO_2_ samples. The spectra were deconvoluted by assuming a Gaussian line shape and Shirley backgrounds by considering Mn 2p doublets with a spin–orbit splitting of 11.5 eV. The deconvolution reveals the presence of Mn in three oxidation states, Mn^2+^, Mn^3+^, and Mn^4+^, suggesting a mixed-valence state and the coexistence of MnO and MnO_2_ on the NaMnO_2_ electrode surface.^34–36^ To gain a deeper understanding of the chemical state of the elements on the electrode, we analyzed the O 1s core-level XPS spectra, as shown in Figure 4c,f,i. For the bare electrode, the deconvoluted spectrum exhibits characteristic peaks corresponding to Mn–O–Mn (529.9 eV) from the NaMnO_2_ host lattice, Mn–OH (532.1 eV), and adventitious adsorbed water H–O–H (534.0 eV),^28,37^ along with a minor Na KLL Auger feature (537.0 eV). Upon immersion and electrochemical cycling in NaNO_3_, Na_2_SO_4_, and Na_2_CO_3_ electrolytes, significant changes occur in the oxygen bonding states. The deconvoluted XPS spectra of both immersed and cycled electrodes exhibit three predominant peaks accompanied by a minor Na KLL Auger feature. The peak at a lower binding energy corresponds to Mn–O–Mn bonds present in the NaMnO_2_ electrode, while the peak within the 531.2–532.4 eV range is associated with Mn–OH and Na–OH species.^37,38^ The peak at a higher binding energy can be attributed to H–O–H species or contributions from the electrolyte.

Electrochemical testing and ex-situ investigation of electrodes after 100 cycles confirmed the presence of two types of pseudocapacitive charge accumulation: diffusion-controlled and surface-controlled. The structural stability of the electrodes during cycling, along with their small particle size, allowed us to achieve good performance under high discharge conditions. A deeper understanding of the electrochemical reasons behind this performance can be obtained by calculating the intercalation/absorption ratio in our electrochemical systems. Biphasic structures of NaMnO_2_, as described in previous studies,^10,39^ were obtained using solid-state reactions with large particles and show a significant dominance of surface-controlled charge accumulation, resulting in relatively low capacity. In our case, smaller particles enable the involvement of near-surface layers of NaMnO_2_, which have an increased interlayer distance, facilitating Na intercalation/deintercalation to a depth of 5–10 nm. This increases the specific capacity without compromising the power density. Surface reactions consist of two components: pseudocapacitive redox reactions and the formation of a thin film on the electrode/electrolyte interface. In the case of Na_2_CO_3_, this film (as confirmed by SEM) forms immediately upon contact with the electrolyte and remains stable during cycling in terms of electrical conductivity (as indicated by the semicircle in Figure 5c), effectively blocking sodium ion intercalation. The low-frequency part of the impedance spectrum exhibits low resistance before and after cycling, indicating a minimal ion diffusion intensity. For Na_2_SO_4_ and NaNO_3_, the high-frequency part of the impedance spectra contains semicircles, indicating higher and more stable resistance after cycling (Figure 5a,b). The slope of the low-frequency part (values between 0.5 and 1) of each impedance spectrum confirms both diffusion- and surface-controlled pseudocapacitive behavior. Linear fitting of the dependencies between CVA cathodic/anodic peaks and the square root of the scan rate (Figure 5d) shows that the calculated *b* values^11^ for the current peaks are within 0.59–0.73. This may indicate that the total current during CVA cycling is the sum of the current related to the slow diffusion-controlled process and the current required to charge the double layer at the electrolyte interface or to initiate fast faradaic reactions on the exposed electrode surface. To quantify the contributions of these two types of capacitance, the Trasatti method^40,41^ has been applied by analyzing the dependencies of specific capacitance and the square root of the scan rate (Figure S4). The calculated diffusion/absorption pseudocapacitance ratio of NaMnO_2_ in different electrolytes is presented in Figure 5i. For the Na_2_SO_4_ electrolyte, we achieve about 55% diffusion-controlled pseudocapacitance contribution, which is higher compared to the results described in refs 42 (44%),^43^ (27%), and^44^ (26%) at high scan rates. This indicates that in our submicrometer electrode particles, we have optimized the intercalation/absorption ratio, and this electrode material is capable of providing the highest electrochemical capacitance and power density.

**Figure 5:**
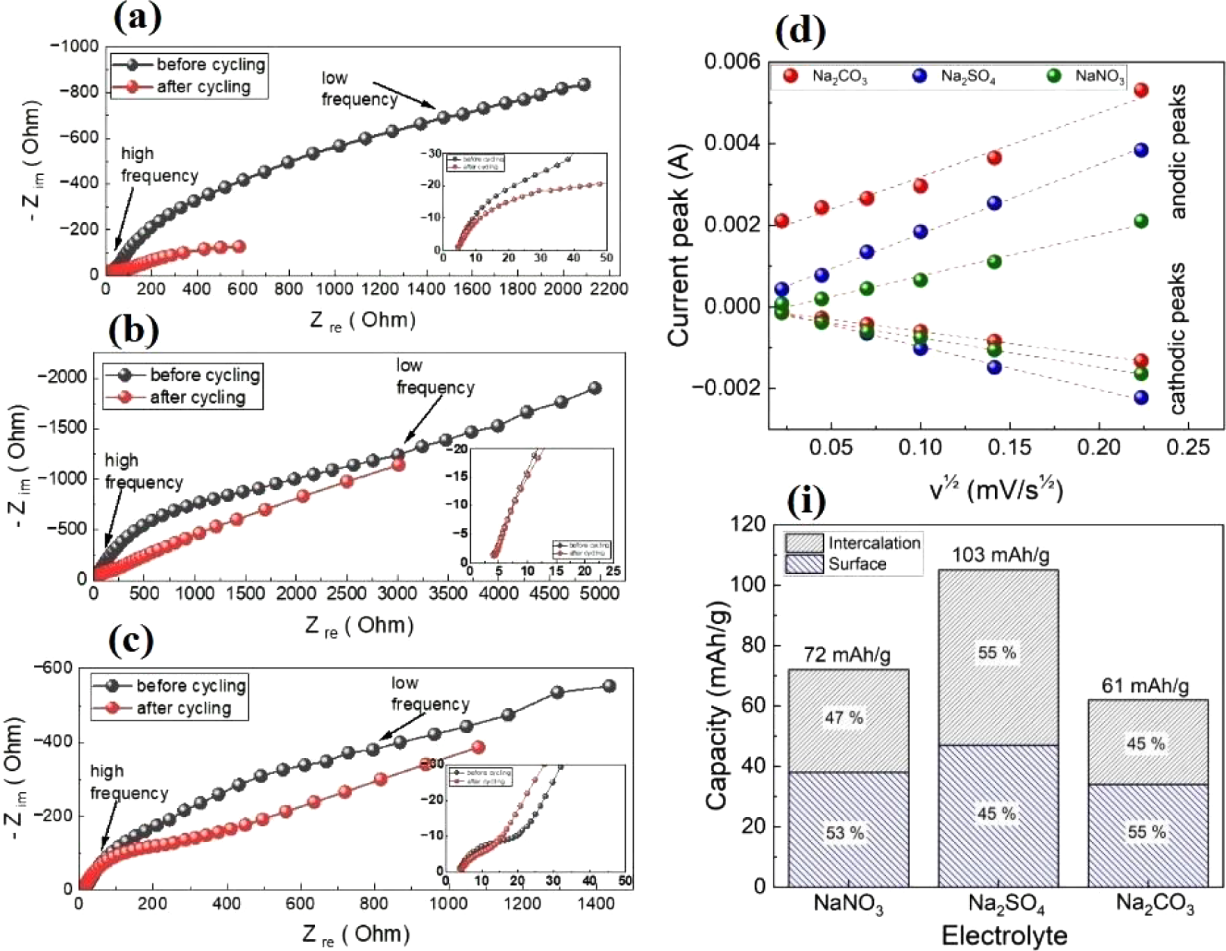
Electrochemical impedance spectroscopy before and after cycling in Na_2_NO_3_ (a), Na_2_SO_4_ (b), and NaCO_3_ (c) electrolytes; the relationship between the cathodic (Ic) and anodic (Ia) current peaks and the square root of the scan rate for different electrolytes (d); (i) calculated contribution of intercalation and surface absorption of ions in different electrolytes.

## Conclusions

The sol–gel-synthesized biphasic NaMnO_2_ exhibits a stable layered structure, ensuring high electrochemical performance in aqueous sodium-ion energy storage applications. The tailored morphology of submicrometer-sized NaMnO_2_ optimizes the diffusion-controlled pseudocapacity contribution, leading to enhanced charge storage efficiency and power density. Electrochemical analysis indicates that the Na_2_SO_4_ electrolyte provides superior cycling stability and capacity retention, while Na_2_CO_3_ induces surface film formation, impeding sodium-ion intercalation. Ex-situ structural investigations (XRD, Raman, TEM, XPS) confirm the material’s stability and surface modification during cycling, revealing a controlled interlayer expansion that contributes to optimized ion storage. Electrochemical impedance spectroscopy and CV analyses validate the optimized intercalation/adsorption ratio (55% and 45%, respectively), which is critical for achieving high power density without compromising capacity. These findings underscore the potential of biphasic NaMnO_2_ as an efficient and scalable cathode material for high-performance sodium-ion batteries and supercapacitors.

## Supplementary Material



## Notes

Detailed information about NaMnO_2_ sample synthesis, interplanar distances extracted from SAED and EDX spectra of electrodes before and after cycling, EDS spectra of fresh and cycled electrodes, and linear fitting of dependencies between capacity and scan rate square for different electrolytes (PDF)
