# Supplementary material for: Enhancement of Diffusion-Controlled Pseudocapacity in Biphasic NaMnO 2 Electrodes for Sodium Batteries by Tailoring Structural and Morphological Properties
Source: ACS Omega. 2025 Jun 25;10(26):28395–403. doi: 10.1021/acsomega.5c03760 (PMC12242681; doi:10.1021/acsomega.5c03760)
Supplement: Supplementary file 1 [file ao5c03760_si_001.pdf]

Supplementary information (SI)

Enhancement of diffusion-controlled Pseudocapacity in Biphasic $NaMnO_2$ Electrodes for Sodium Batteries by tailoring Structural and Morphological properties

Andrii Boichuk[1,2], Tetiana Boichuk[1*], Marie Krečmarová[1], Mahesh Eledath Changarath[1], Rafael Abargues[1], Said Agouram[3], Juan F. Sanchez-Royo[1†]

[1] ICMUV, Instituto de Ciencia de Materiales, Universidad de Valencia, 46071 Valencia, Spain
[2] King Danylo University, 76000, Ivano-Frankivsk, Ukraine
[3] Department of Applied Physics and Electromagnetism, University of Valencia, 46100 Valencia

[*]Corresponding author: Tetiana.Boichuk@uv.es (Tetiana Boichuk)
[†]Corresponding author: Juan.F.Sanchez@uv.es (Juan F. Sánchez-Royo)

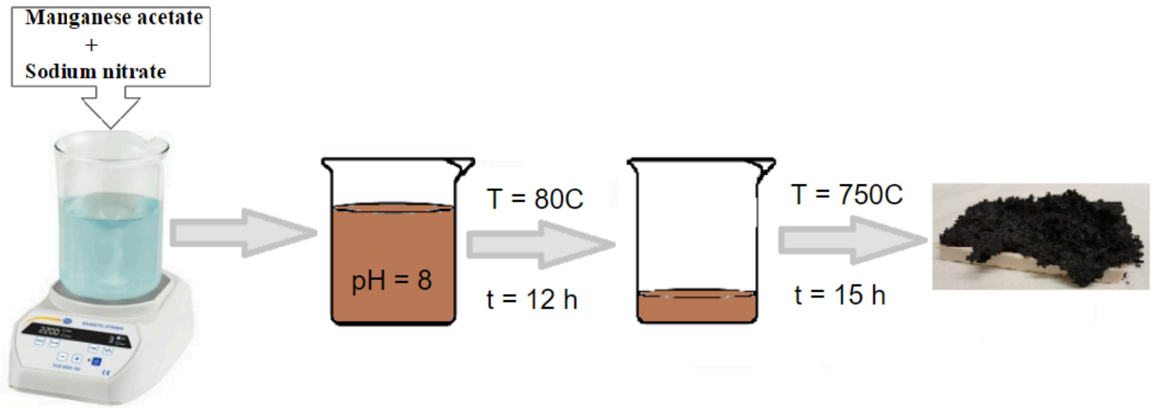

Figure S1. Step by step synthesis of $NaMnO_2$ material

$NaMnO_2$			
before cycling		***after cycling***	
d_{hkl} (nm)	(hkl)/ phases	d_{hkl} (nm)	(hkl)/ phases
0.65	(001) ortho	0.8-1	(001) ortho
0.54	(001) mono	0.8-1	(001) mono
0.24	(200)/(110)ortho &/or (-111)/(-202) mono	0.24	(200)/(110)ortho &/or (-111)/(-202) mono

0.21	(111) mono	0.21	(111) mono
0.14	(-400)/(-313)/<br>(-204)/(020)/(-402)/<br>(-401) mono	0.14	(-400)/(-313)/<br>(-204)/(020)/(-402)/<br>(-401) mono

Table S1. Interplanar distances extracted from SAED before and after cycling.

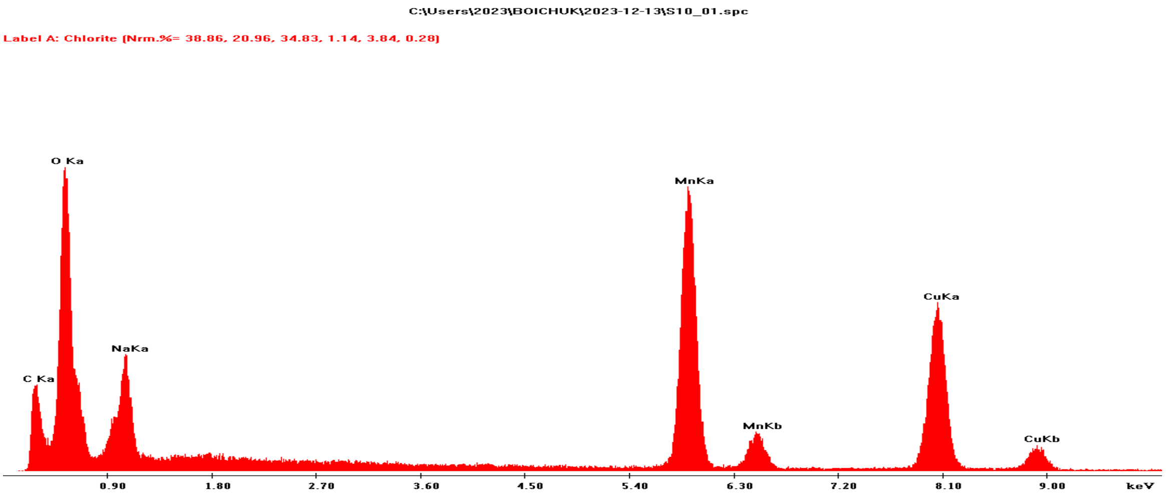

Figure S2. EDX spectra of the electrode after cycling

Sample	Mn (at. %)	Na (at. %)	Mn/Na ratio
before cycling	70.2 ± 1.5	29.8 ± 2.5	2.36
after cycling	67.9 ± 1.5	32.1 ± 2.5	2.11

Table S2. Percentage of components in electrodes before and after cycling based on EDX spectra

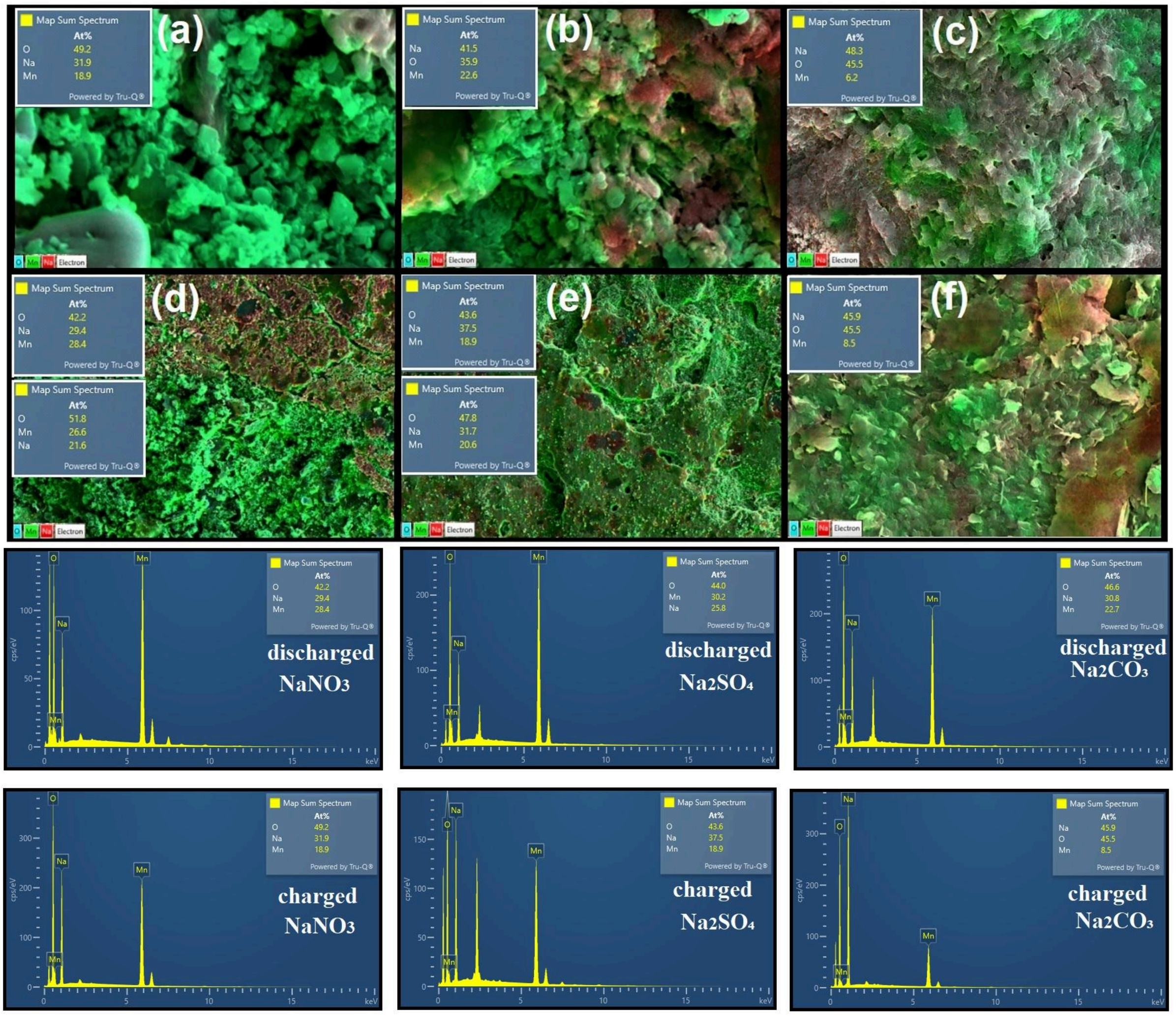

Figure S3. SEM map after cycling in $NaNO_3$ (a,d), Na_2SO_4 (b,e), and Na_2CO_3 (c,f) electrolyte, respectively and corresponding EDS spectra.

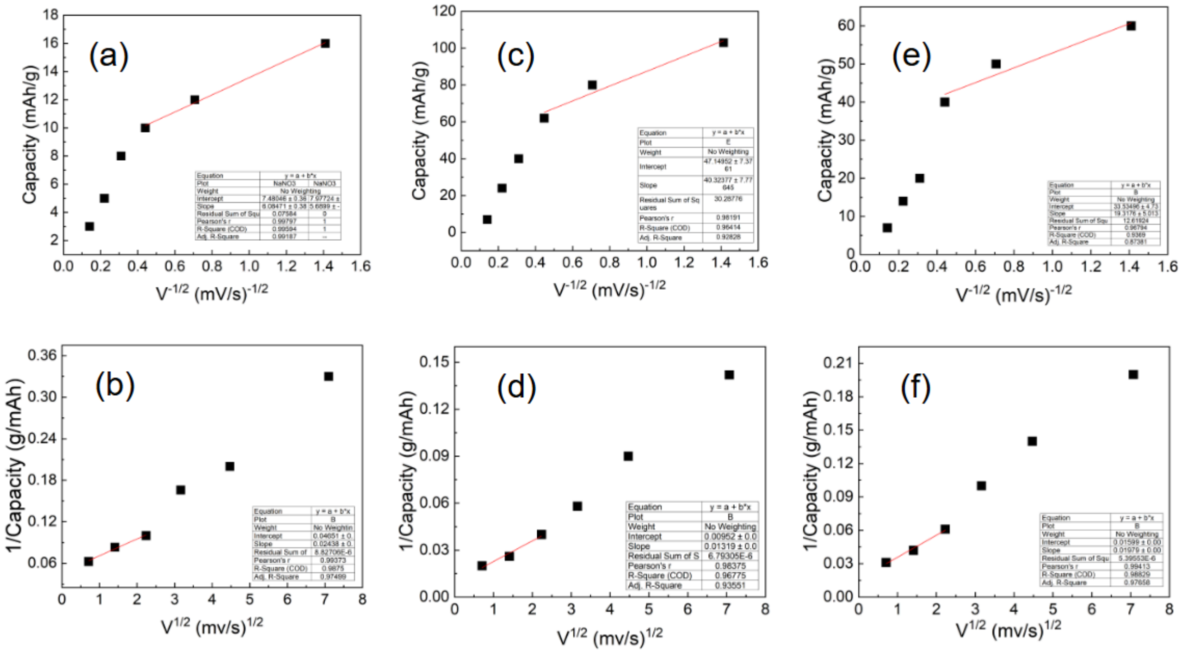

Figure S4. Linear fitting of dependencies between capacity and scan rate square for different electrolytes: $NaNO_3$(a,b), Na_2SO_4 (c,d), and Na_2CO_3 (e,f) electrolytes respectively
